# Simplifying radiology reports with large language models: privacy-compliant open- versus closed-weight models

**DOI:** 10.1007/s00330-026-12329-6

**Published:** 2026-02-12

**Authors:** Annemarie Katharina Proff, Babak Salam, Mohammed Hayawi, Dmitrij Kravchenko, Narine Mesropyan, Taraneh Aziz-Safaie, Tatjana Dell, Maike Theis, Claus Christian Pieper, Alois Martin Sprinkart, Daniel Kütting, Julian Alexander Luetkens, Sebastian Nowak, Alexander Isaak

**Affiliations:** 1 Department of Diagnostic and Interventional Radiology, University Hospital Bonn, Bonn, Germany; 2 Quantitative Imaging Lab Bonn (QILaB), University Hospital Bonn, Bonn, Germany; 3 Department of Diagnostic and Interventional Radiology, University Hospital Aachen, Aachen, Germany

**Keywords:** Artificial intelligence, Large language model, Open-source software, Patient communication, Radiology

## Abstract

**Objectives:**

Large language models (LLMs) like generative pre-trained transformer (GPT) can simplify radiology reports for medical laypersons, but privacy concerns limit their clinical applicability. This study compares closed-weight and in-hospital deployed privacy-compliant open-weight LLMs in generating patient-friendly radiology reports.

**Materials and methods:**

A total of 60 radiology reports containing indication and impression sections (15 each from X-ray, ultrasound, CT, and MRI) were translated into lay-friendly versions using different LLMs: one commercial closed-weight model (GPT-4o) and two in-hospital deployed open-weight models (Llama-3-70b, Mixtral-8x22B). All reports were evaluated for readability (Flesch reading ease, reading time, word and sentence count). 21 medical laypeople assessed understandability using a 5-point Likert scale. Linear mixed-effects models and H-Kruskal–Wallis test were used for statistical analysis.

**Results:**

LLM-generated reports demonstrated significantly improved readability, achieving higher Flesch reading ease scores (GPT-4o: 46 ± 7, Llama-3-70b: 44 ± 6, Mixtral-8x22B: 44 ± 6, original: 17 ± 13; *p* < 0.001). All three LLM reports yielded markedly higher layperson-understandability ratings than the original reports (GPT-4o: 4.4 ± 0.1; Llama-3-70B: 4.3 ± 0.1; Mixtral-8x22B: 4.1 ± 0.1 vs. 1.5 ± 0.1; *p* < 0.001 for each), with no significant difference between GPT-4o and Llama-3-70B (*p* = 0.136). Mixtral-8x22B and Llama-3-70B produced more errors with potential for patient harm than GPT-4o (*p* = 0.005 and *p* = 0.025, respectively). Imaging modality did not influence understandability (all *p* > 0.05).

**Conclusion:**

LLMs substantially improved layperson understanding of radiology reports. Open-weight, on-premises LLMs like Llama-3-70B show strong potential for real-world clinical use, though human oversight is still required.

**Key Points:**

***Question*** *Can locally deployed open-weight large language models (LLMs) improve the readability and understandability of radiology reports for medical laypersons at a level comparable to closed-weight models?*

***Findings*** *LLMs significantly improved quantitative readability scores and qualitative ratings of layperson understandability; Llama-3-70B and GPT-4o showed comparable performance, and although the open-source models exhibited a higher error rate, they still performed well overall.*

***Clinical relevance*** *Open-weight LLMs provide a privacy-compliant way to generate a template for patient-friendly radiology reports suitable for real-world clinical use.*

**Graphical Abstract:**

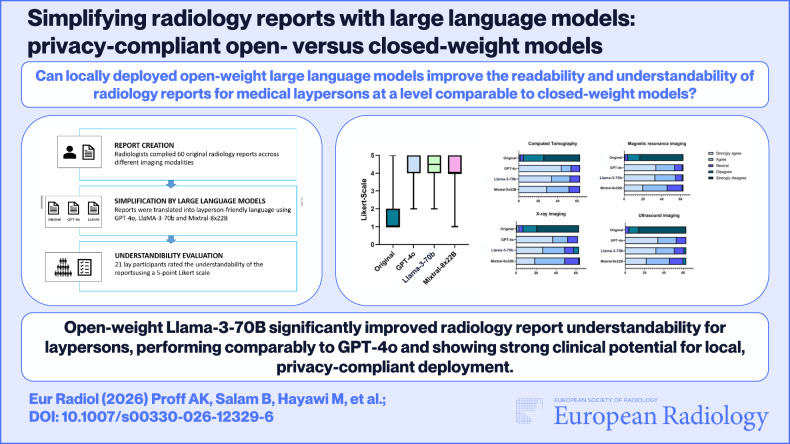

## Introduction

Clear communication of radiological findings is essential to support patient understanding and reduce uncertainty [1]. However, conventional radiology reports are typically intended for referring physicians and often contain specialized medical and technical terminology that can be difficult for medical laypersons to understand [2–5]. Several studies suggest that large language models (LLMs) can assist in generating simplified, layperson-friendly radiology reports, offering a promising opportunity to enhance patient communication [6]. Closed-weight models (proprietary models with confidential architectures and weight) such as GPT-4 have already been evaluated in different tasks processing medical reports, such as error detection, and the capability to reliably translate radiological findings into language accessible to laypersons [7–12]. Strict data protection regulations in many countries often hinder the use of cloud-based LLMs, creating significant hurdles for processing patient data outside secure clinical networks. Given these regulations, on-premises, privacy-compliant deployments of open-weight models are the most feasible way to retain data control [13].

Previous studies have shown that open-weight models are suitable for various tasks [14–16]. Simplified, patient-friendly reporting formats could help to improve understanding and patient–physician communication [17–20]. However, despite the widespread use of algorithmic readability scores, few studies have incorporated human participants and real layperson feedback to assess the understandability of LLM-generated medical texts. Therefore, there is a need for more human-centered evaluation methods alongside automated metrics, especially in the context of patient communication, building on and extending previous work that focused solely on objective parameters [15, 21].

This study aims to compare the performance between a closed-weight model (GPT-4o) and open-weight models (Llama-3-70b and Mixtral-8x22B) in simplifying radiology reports for medical laypersons across different imaging modalities.

## Materials and methods

After the data privacy consultation, a specific approval from the institutional review board was not required, as no patient information was used in this study.

### Preparation of radiological reports

60 fictional radiology reports, based on realistic clinical scenarios and aligned with original findings, containing indication and impression sections, were created in German language by two radiologists (B.S., A.I.). The reports encompassed four imaging modalities: CT, MRI, X-ray, and ultrasound (15 reports per modality). All reports followed a standardized, professional format according to general clinical practice.

### Prompt development and selection

We developed a single prompt to generate layperson radiology summaries at approximately eighth-grade reading level, preserving key clinical content and enforcing a five-part structure (examination, reason, findings, simple explanation, impression). The wording was iteratively refined on 12 exemplar reports and reviewed by two board-certified radiologists to emphasize structural adherence, first-mention explanation of medical terms, and avoidance of new diagnostic statements. The final prompt was used unchanged as a zero-shot instruction for all models; no domain fine-tuning or systematic prompt optimization was performed. The final prompt is provided in Supplement S1.

### Generating explained reports using large language models

**Fig. 1:**
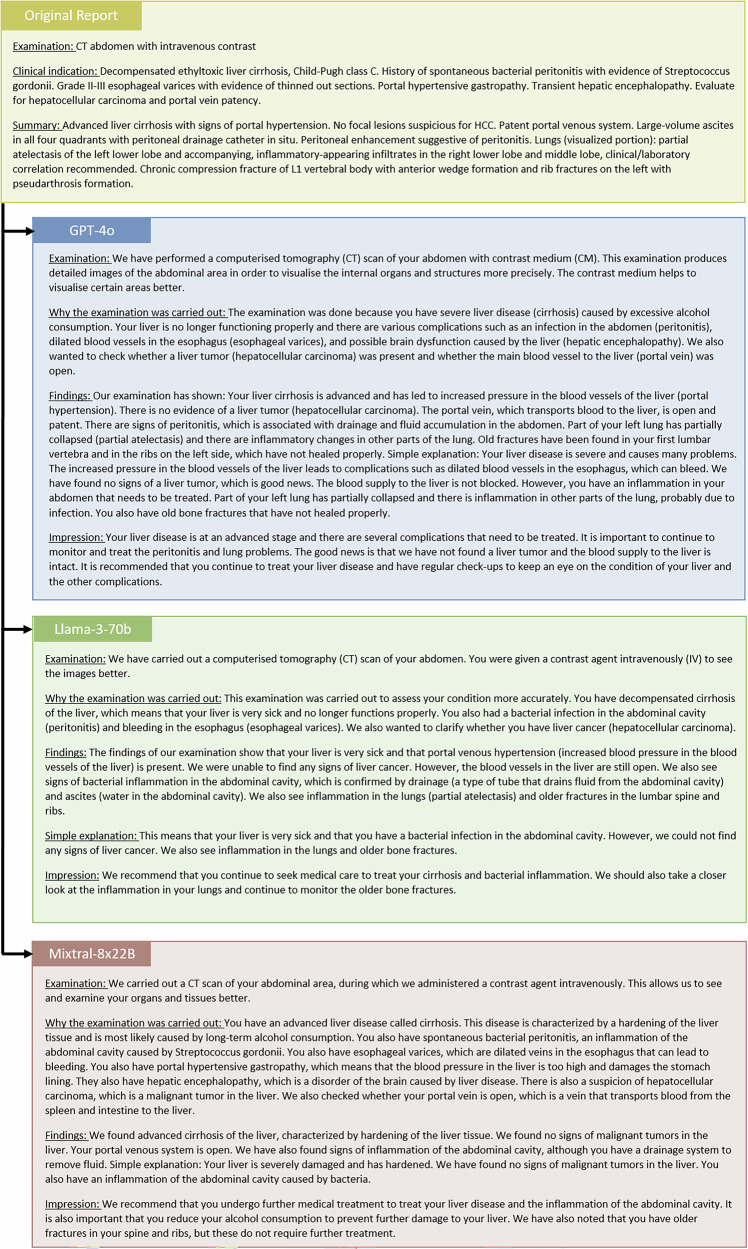
Exemplary original radiology report (translated from German) alongside its simplified versions generated by three large language models. The English renderings were produced by a human translator (native-speaking author). HCC, hepatocellular carcinoma

Each report was processed by three LLMs: the closed-weight GPT-4o (OpenAI, version June 2024), the open-weight Llama-3-70b (open-weight, Meta, version June 2024), and the open-weight Mixtral-8x22B (Mistral AI, version June 2024). For both open-weight models (Llama-3-70b and Mixtral-8x22B), the officially released weights without any alterations by domain-specific fine-tuning were used. Open-weight models were deployed on hospital-controlled, on-premises servers. To generate the simplified reports, we used an open-source inference software (vLLM v6.0.1) on eight NVIDIA A100 80GB GPUs. An exemplary original report, along with the corresponding simplified reports from all three LLMs, is provided in Fig. 1. In total, 180 simplified outputs were generated (60 original reports × 3 models). The chat session was restarted before each repetition of the prompt to prevent any potential bias in the generated response via cached tokens. Exemplary reports and corresponding outputs can be found in Supplement 2–5.

### Error analysis

Two radiologists performed a consensus review of the LLM-generated reports (A.P., A.I.). Errors posing potential critical harm to patients and formal inaccuracies were identified. Each of the 60 reports was evaluated per model and coded as either containing an error or not containing an error, based on the presence of at least one error of the respective category. Errors with potential for patient harm were defined as critical errors, like misdiagnoses (e.g., hepatocellular carcinoma instead of liver metastases) or incorrect explanations of facts that indicate a disease pattern of another entity or aggravate the disease. We defined formal inaccuracies as errors that do not change disease interpretation or clinical management.

### Questionnaire design

Questionnaires were designed to evaluate the general understandability of the original report and the LLM-generated reports on a Likert scale (from 1 “strongly disagree” to 5 “strongly agree”) by medical laypersons. Of the 60 reports created, five questionnaires were compiled, each containing 12 report sets with three reports for each imaging modality (CT, MRI, X-ray, and ultrasound). Each report set included four different report versions (original, GPT-4o, Llama-3-70b, and Mixtral-8x22B), resulting in 48 report evaluations per participant. To minimize sequence and anchoring effects, we used two-level randomization. Sixty reports were split into five questionnaire versions (12 sets each), randomly assigned to participants. For each participant, both the order of report sets and the four versions within each set (Original, GPT-4o, Llama-3-70B, Mixtral-8x22B) were randomized. Participants were blinded to the generating model and rated only the understandability.

### Readability analysis

The estimated reading time was calculated based on the total word count of the text and an assumed average reading speed of approximately 230 words per minute. This value reflects the average reading pace of an adult for standard German-language texts. To objectively assess the readability of texts, Flesch reading ease score adapted for the German language was applied. This metric evaluates textual readability based on average sentence length and word complexity, expressed as syllables per word. The score is directly linked to educational levels and allows classification of texts according to the approximate school grade required for comprehension. The Flesch reading ease scores were interpreted using standard thresholds: 90–100 = very easy (primary school level), 80–90 = easy, 70–80 = fairly easy, 60–70 = standard (7th–9th grade), 50–60 = fairly difficult (high school level), 30–50 = difficult (college level), and 0–30 = very difficult (academic or technical texts). The score has already been established in previous studies [22].

### Statistical analysis

Statistical analyses were conducted using Prism (version 10.0.2; GraphPad Software Inc.), SPSS Statistics (version 29 IBM), R (version 4.4.1) and R Studio (version 2025.05.1 Build 513, Posit Software). Normality was assessed visually and with the Shapiro–Wilk test. Continuous variables are reported as mean ± standard deviation; ordinal variables as median and interquartile range (IQR). Readability parameters were compared using Kruskal–Wallis tests. Understandability ratings were analyzed with linear mixed-effects models including report ID and participant ID as random intercepts. Robustness was checked using Fisher’s exact tests per report with Bonferroni–Holm correction. Differences in error rates across LLMs were examined with Friedman and Wilcoxon signed-rank tests. Krippendorff’s alpha was used to measure inter-rater reliability. A value between 0.61 and 0.80 indicates moderate to substantial agreement, while a value between 0.81 and 1.00 suggests strong agreement among raters. Friedman test and Wilcoxon signed-rank test were conducted to examine differences in error rates across the three LLMs. Statistical significance was defined as *p* < 0.05.

## Results

### General report parameters

The estimated reading time for original reports was significantly shorter (15.2 ± 5.4 s) compared to LLM-generated reports (GPT-4o: 71.4 ± 12.6 s; Llama-3-70b: 64.3 ± 8.2 s; Mixtral-8x22B: 72.5 ± 12.3 s; each *p* < 0.001 vs. original). Word count was lowest for original reports (56 ± 20 words) and highest for Mixtral-8x22B (275 ± 48 words), followed by GPT-4o (269 ± 49 words) and Llama-3-70b (244 ± 31 words); *p* < 0.001 for all LLM-generated reports compared to the original versions.

### Quantitative readability analysis

Original radiology reports had a significantly lower Flesch reading ease score (17.1 ± 12.8) compared to all LLM-generated versions: 45.9 ± 7.0 for GPT-4o, 43.7 ± 6.3 for Llama-3-70b (all *p* < 0.001 vs. original), and 44.2 ± 6.4 for Mixtral-8x22B. No statistically significant differences in Flesch reading ease score were observed among the LLMs (GPT-4o vs. Llama-3-70b: *p* = 0.174; GPT-4o vs. Mixtral-8x22B: *p* = 0.252; Mixtral-8x22B vs. Llama-3-70b: *p* = 0.830); see Tables 1 and 2.

**Table 1:** General readability and structure characteristics of original and simplified radiology reports generated by large language models

	Original (*n* = 60)	GPT-4o (*n* = 60)	Mixtral-8x22B (*n* = 60)	Llama-3-70b (*n* = 60)	*p*-value
Flesch reading ease	17 ± 13	46 ± 7	44 ± 6	44 ± 6	< 0.001
Estimated reading time (s)	15.2 ± 5.4	71.4 ± 2.6	72.5 ± 2.4	64.3 ± 8.2	< 0.001
Characters	522 ± 178	1994 ± 360	2047 ± 369	1790 ± 226	< 0.001
Words	56 ± 20	269 ± 49	275 ± 48	244 ± 31	< 0.001
Sentences	12 ± 3	23 ± 4.0	22 ± 4	17 ± 3	< 0.001

**Table 2:** Pairwise comparison of readability and structural metrics of simplified radiology reports between different large language models

	GPT-4o (*n* = 60)	Mixtral-8x22B (*n* = 60)	Llama-3-70b (*n* = 60)	*p*-value GPT-4o vs. Llama-3-70b	*p*-value GPT-4o vs. Mixtral-8x22B	*p*-value Llama-3-70b vs. Mixtral-8x22B
Flesch reading ease	46 ± 7	44 ± 6	44 ± 6	0.174	0.252	0.830
Estimated reading time (s)	71.4 ± 2.6	72.5 ± 2.4	64.3 ± 8.2	0.018	0.018	0.007
Characters	1994 ± 360	2047 ± 369	1790 ± 226	0.016	0.574	0.003
Words	269 ± 49	275 ± 48	244 ± 31	0.033	0.011	0.008
Sentences	23 ± 4.0	22 ± 4	17 ± 3	< 0.001	0.603	< 0.001

### Qualitative understandability analysis

**Fig. 2:**
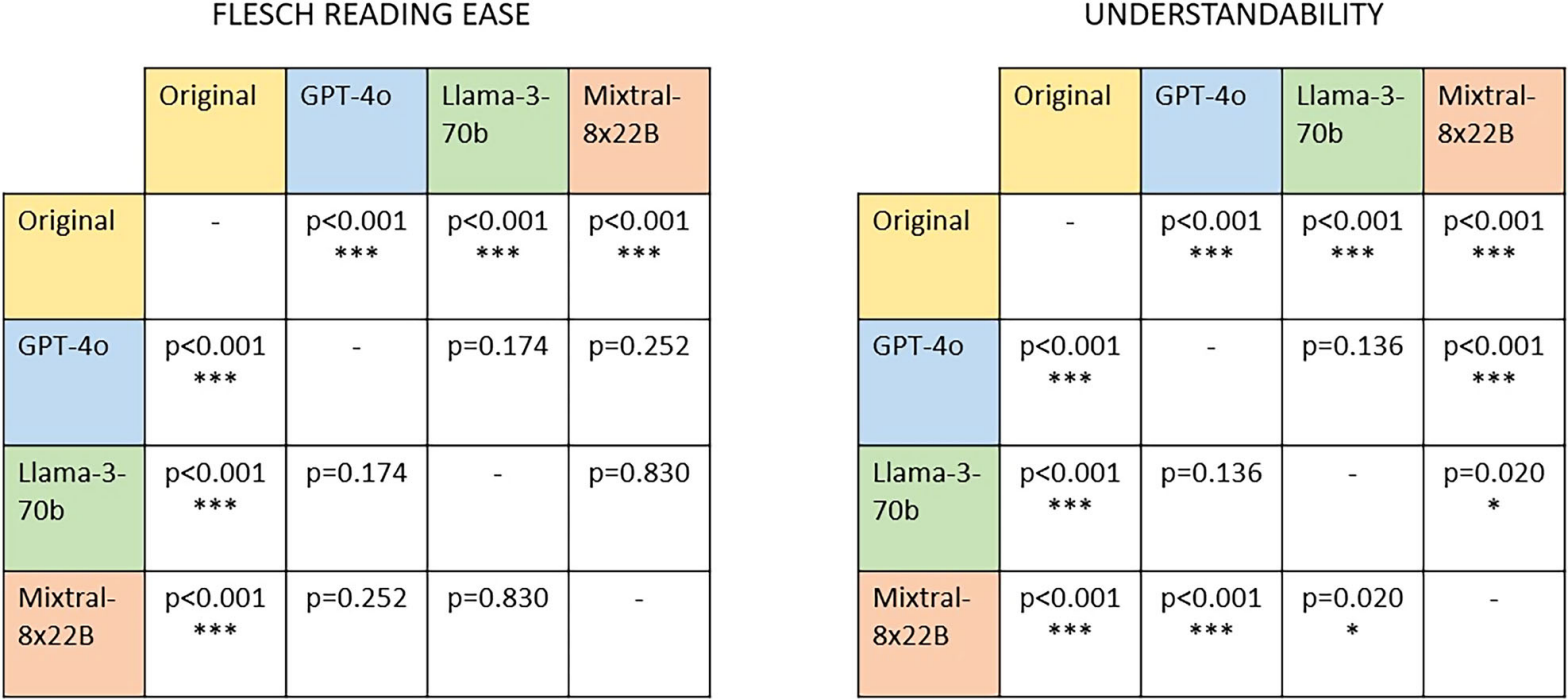
Pairwise comparison matrix of readability metrics across original reports and large language models-generated versions. The left panel displays differences in the objective Flesch reading ease index (H-Kruskal–Wallis Test); the right panel shows differences in subjective understandability ratings by medical laypersons (linear mixed-effects model, Bonferroni–Holm correction). Off-diagonal cells indicate pairwise *p*-values; Significance markers: *p* < 0.05; * *p* < 0.01; ** *p* < 0.001

**Fig. 3:**
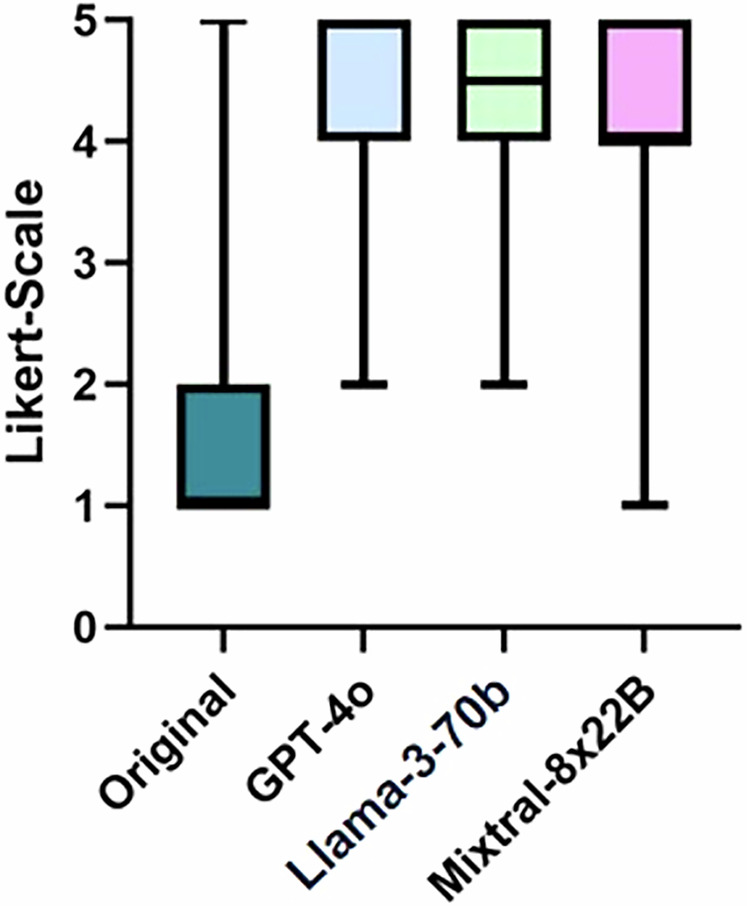
Box-and-whisker plots display layperson-understandability ratings for the original radiology reports and LLM-generated versions (Likert scale: 1 = strongly disagree, 5 = strongly agree). Medians are: original = 1, GPT-4o = 5, Llama-3-70B = 4.5, Mixtral-8x22B = 4

**Fig. 4:**
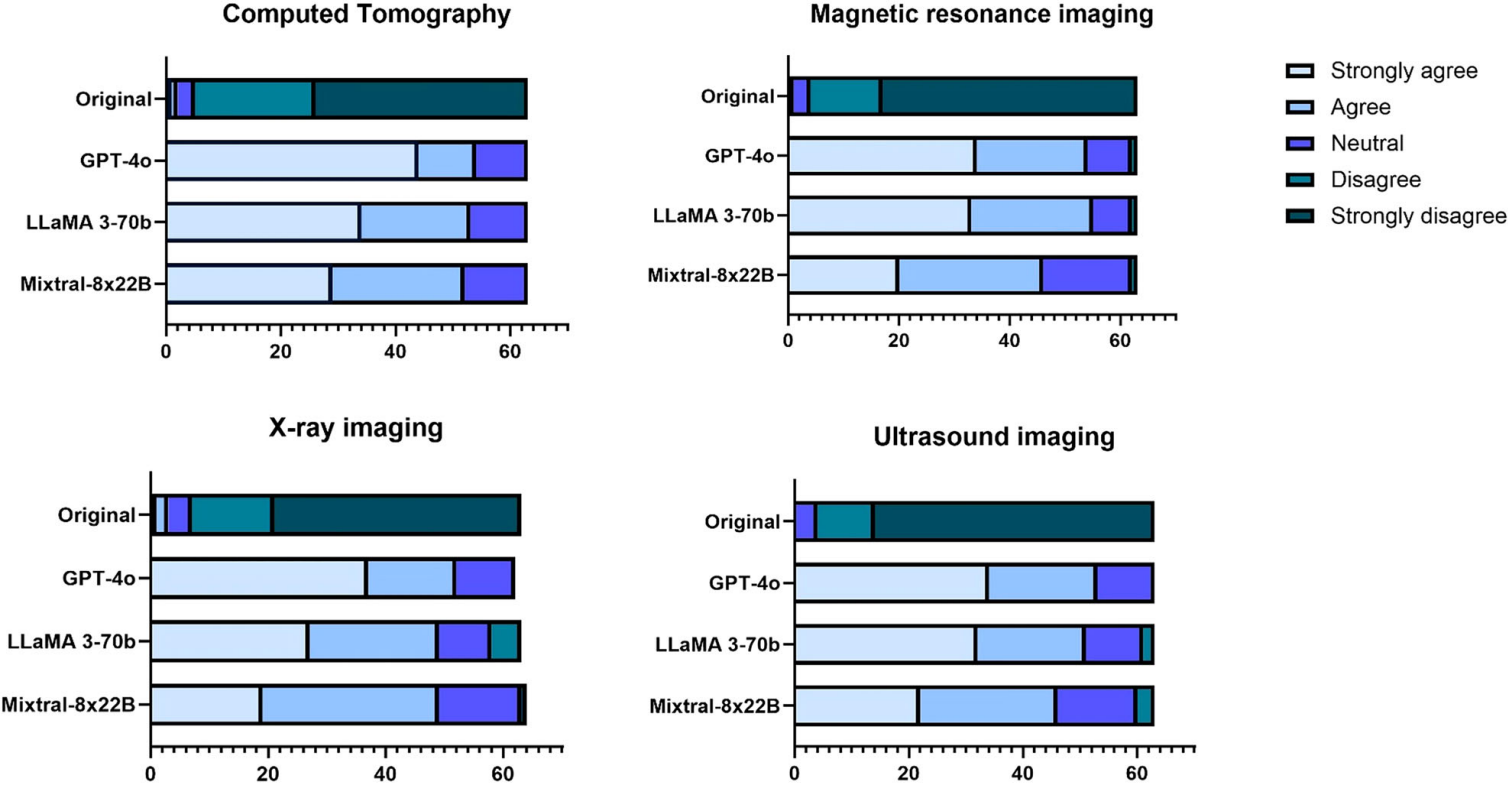
Bar graphs show the distribution of layperson-understandability ratings across four imaging modalities (CT, MRI, X-ray, ultrasound) for original reports and large language models-generated versions

In total, 21 medical laypersons participated in this study. The majority of participants were younger than 50 years (18/21; 85.7%), while a smaller proportion belonged to an age group over 50 years (3/21; 14.3%). 8/21 participants were female (38.1%). 11/21 participants held an academic degree (52.4%), whereas 10/21 did not (47.6%). The average time required to complete the questionnaire was a median of 64.5 min (IQR 32.5–73.0). There was a non-significant trend that participants with an academic degree gave higher readability rating scores (3.70 ± 0.12) compared to those without an academic degree (3.33 ± 0.15; *p* = 0.073). GPT-4o received the highest average score for layperson understandability (4.4 ± 0.1; *p* = 0.136 compared to Llama-3-70b, *p* < 0.001 compared to Mixtral-8x22B), followed by Llama-3-70b (4.3 ± 0.1) and Mixtral-8x22B (4.1 ± 0.1), with all models performing significantly better than the original reports (1.5 ± 0.1; *p* < 0.001 for all comparisons with LLM models); see Figs. 2, 3 and Table 3. In the extended linear mixed model the type of modality including CT (median 4 [IQR 2–5]), MRI (median 4 [IQR 2–5]), X-ray (median 4 [IQR 2–5]), and ultrasound (median 4 [IQR 1–5]) had no significant effect on the understandability ratings of translated reports (e.g., CT vs. MRI: *p* = 0.932; CT vs. x-ray: *p* = 0.923; CT vs. ultrasound: *p* = 0.841; MRI vs. x-ray: *p* = 1.000; MRI vs. ultrasound: *p* = 0.996; x-ray vs. ultrasound: *p* = 0.997). Inter-rater reliability indicated a substantial level of inter-rater reliability (Krippendorff’s alpha: 0.71), see Fig. 4.

**Table 3:** Understandability ratings of radiology reports by medical laypersons (*n* = 21) assessed using a five-point Likert scale (1—strongly disagree, 5—strongly agree)

Report	Original reports	GPT-4o	Llama-3-70b	Mixtral-8x22B
Median	1	5	4.5	4
Q1	1	4	4	4
Q3	2	5	5	5
Mean	1.5	4.4	4.3	4.1
SD	0.7	0.7	0.8	0.8
Min	1	2	2	1
Max	5	5	5	5

### Error analysis

Mixtral-8x22B had the highest rate of errors with potential for patient harm (8 critical harm errors in 6/60 reports [10%]), followed by Llama-3-70b (5 critical harm errors in 5/60 reports [8.3%]), and GPT-4o (no critical harm errors). Pairwise comparisons indicated that Mixtral-8x22B and Llama-3-70b produced significantly more errors with potential for patient harm than GPT-4o (*p* = 0.005 and *p* = 0.025, respectively); there were no significant differences between Llama-3-70b and Mixtral-8x22B (*p* = 0.317). Formal inaccuracies (without potential harm for the patient) were also most common in Mixtral-8x22B (Mixtral-8x22B: 4 errors, Llama-3-70b: 2 errors, GPT-4o: 1 error; *p* = 0.36).

## Discussion

This study compares closed-weight and privacy-compliant open-weight LLMs for generating patient-friendly radiology reports. All evaluated models substantially improved layperson understanding; Llama-3-70B performed comparably to GPT-4o, highlighting its potential for privacy-preserving local deployment. Nonetheless, the open-weight models (Llama-3-70B, Mixtral-8x22B) showed higher rates of errors with potential for patient harm than GPT-4o, reinforcing the value of clinician oversight.

Whereas previous studies primarily examined proprietary models, mainly from OpenAI’s GPT family, the present study additionally evaluates open-weight alternatives that were deployed on local hospital servers [7, 10, 18]. Our results are in line with previous benchmark tests and studies that showed high performance of GPT-4o in simplifying medical text [10]. Interestingly, the Llama-3-70b model showed no statistically significant differences in readability scores and understandability ratings in our study, suggesting that open-weight models can achieve comparable performance to closed models in simplifying reports. Recent studies also demonstrate non-inferiority of open-weight to closed-weight models in other medical tasks [14, 23–25].

While all LLMs significantly improved the Flesch reading ease score of the reports, raising the average from academic level in original reports to values of high school level and simpler literacy levels, no significant differences in Flesch reading ease were found among the three tested LLMs. The resulting Flesch reading ease scores of the LLM reports mostly remained in the range of 30–50, corresponding to upper secondary to college-level difficulty. Interestingly, qualitative understandability scores based on layperson ratings did differ, indicating that readability metrics alone may not fully capture a text’s comprehensibility [18]. While the Flesch reading ease score accounts for sentence and word length, it does not consider semantic clarity or contextual cues, which likely influence perceived understanding more strongly [22]. These metrics do not account for semantic clarity, contextual coherence, or the presence of domain-specific terminology, which are essential for genuine understanding, particularly among individuals without medical training [26, 27]. For example, a report may score well on readability due to short sentences but still be incomprehensible to lay readers if it contains unexplained medical jargon or lacks logical flow. Conversely, a text with longer sentences may be more understandable if it provides clear explanations and contextual information. This highlights the necessity of including human-centered evaluation alongside algorithmic metrics, particularly in the context of patient communication and extends previous studies investigating the objective reading parameters [15, 21]. Previous GPT-4–based studies using layperson ratings found that simplified radiology reports improved patient understanding and satisfaction [28, 29].

In the present study, the type of imaging modality had no significant influence on understandability ratings. Other studies that analyzed simplified radiology reports across different imaging modalities reached similar conclusions [15]. This indicates that LLM performance is robust across different radiology domains, including CT, MRI, ultrasound, and X-ray, which supports their broad applicability.

Despite the overall positive results regarding understandability, potential risks associated with LLM-generated content must be critically considered [30]. Cloud-based LLM services in clinical workflows raise distinct privacy and data-governance concerns, because protected health information is transmitted to third-party infrastructure and may be retained for service improvement [31–33]. Beyond regulatory constraints, recent work has shown that large models can memorize and later expose sensitive content or be prompted to reveal private information [34]. To mitigate these risks, the literature recommends rigorous de-identification, privacy-preserving training or inference, segregated retrieval layers, locally hosted models, and human verification of patient-facing outputs [32, 33]. These considerations informed our decision to evaluate open-weight, on-premises LLMs as a more privacy-preserving alternative.

Furthermore, even highly capable models such as GPT-4o or Llama-3-70b are not immune to so-called hallucinations that can lead to inaccuracies, misleading simplifications or even to trust-breaking errors that could undermine patients’ confidence in the information provided. These may include incorrect downplaying of findings (e.g., referring to a tumor as “small”), omission of clinically relevant information, or ambiguous descriptions of procedures. In real-world clinical settings, such errors could lead to unnecessary anxiety, unrealistic expectations, or reduced treatment adherence. The generated simplifications in this study showed errors with potential for patient harm in 10% of Mixtral-8x22B reports, 8.3% of Llama-3-70b reports, and in none of GPT-4o reports. Open-weight models show potential but require further refinement (e.g., domain-tuning, safety guardrails, human-in-the-loop validation). Nevertheless, despite the current performance gap, open models remain essential for privacy-preserving, on-premises deployment of clinical tasks. Our findings underscore that LLM-generated summaries should be used cautiously as a complementary tool, not a replacement for physician oversight. While our primary objective was to compare zero-shot behavior of closed- and open-weight models under equivalent conditions, the higher rate of potentially harmful errors in some open-weight models underscores the need for mitigation before clinical use. In this study, we restricted mitigation to iterative prompt refinement during study design to reduce avoidable hallucinations and enforce safety-oriented phrasing, and deliberately did not implement systematic pipelines within the evaluation [35, 36]. For future prospective use, especially of open-weight models, simple post-processing steps could further reduce critical errors while preserving the benefits of local deployment (privacy, data governance, configurability). Examples include structured prompting and template-based outputs to constrain free text, explicit reasoning and verification prompts to check each factual claim against the source report, automated second-pass verification (e.g., chain-of-verification procedures), and cross-model agreement checks that flag disagreements for clinician review [37]. A human-in-the-loop sign-off before any patient-facing communication remains essential, and these layered mitigations together offer a pragmatic path to more reliable and safer deployment of open-weight models [38].

The results of our study, demonstrating comparable performance between GPT-4o and Llama-3-70b in generating layperson-friendly reports, underscore the growing potential of open-weight models to compete with proprietary models and support for their integration into hospital workflows [39].

Prompt wording is a well-recognized determinant of generative model behavior and can substantially influence factuality, style, and risk of hallucination [40]. Differences in safety-critical error rates between models observed in our study could therefore reflect differential sensitivity of model families to the specific zero-shot instructions rather than intrinsic capability differences alone [35]. Because we used a single, clinically motivated zero-shot prompt, our results reflect performance under this one prompt rather than inherent model safety. Other prompts and multi-layer mitigation (templates, verification, human review) may further reduce critical errors and should be tested before clinical use [35, 41].

This study has several limitations. First, the study design relied on subjective rating scales to assess understandability, which remain prone to individual variability and potential bias. Second, given the rapid pace of LLM development (with new versions emerging on a near-weekly basis), we were unable to replicate our experiments for models released after the recruitment period. However, we believe that these insights comparing open- vs. closed-weight models for report simplification also largely carry over to the latest model generations. Furthermore, the models evaluated (GPT-4o, Mixtral-8x22B, and Llama-3-70b) were tested using zero-shot static outputs without iterative refinement or clinician supervision, which may not reflect future implementations incorporating user feedback or domain-specific fine-tuning. Because LLM-generated findings had a distinctive explanatory style, laypersons could not be fully blinded; however, they remained unaware of which model produced them. While randomization at both the report set and intra-set levels was implemented to minimize ordering bias, minor sequence effects cannot be entirely excluded.

We did not perform domain-specific fine-tuning of the open-weight models. While local fine-tuning could plausibly improve clinical accuracy and reduce semantic errors, it requires curated labeled data, introduces the risk of over-fitting to institutional language patterns, and may increase memorization of sensitive information if training data are not thoroughly de-identified. Future work should evaluate the benefits and safety implications of domain adaptation under robust privacy safeguards. Finally, our findings are based on German-language reports and may not generalize to other languages, especially as LLMs typically perform best in English [42, 43]. Future prospective clinical studies with larger, more diverse cohorts and multimodal sets are needed to validate and extend these findings in clinical practice.

In conclusion, our findings highlight the significant potential of open-weight LLMs as an in-hospital deployed and privacy-compliant alternative for simplifying radiological reports. The local deployment of these models within clinical workflows offers a promising approach for generating layperson-friendly findings without compromising data protection regulations. While continued development is necessary to further improve model accuracy, the advancement of high-performing LLMs marks an important step toward enhancing clinician–patient communication. Going forward, a multidisciplinary approach involving clinicians, AI researchers, and ethicists will be crucial to ensure these tools are safely and effectively integrated into healthcare, ultimately improving patient care.

## Supplementary information

Supplementary information

## Abbreviations

CT: Computed tomography
GPT: Generative pre-trained transformer
IQR: Interquartile range
LLMs: Large language models
MRI: Magnetic resonance imaging

## References

[1] Edgman-Levitan S, Schoenbaum SC (2021) Patient-centered care: achieving higher quality by designing care through the patient’s eyes. Isr J Health Policy Res 10:21 33673875 10.1186/s13584-021-00459-9 PMC7934513

[2] Gunn AJ, Gilcrease-Garcia B, Mangano MD, Sahani DV, Boland GW, Choy G (2017) JOURNAL CLUB: Structured feedback from patients on actual radiology reports: a novel approach to improve reporting practices. AJR Am J Roentgenol 208:1262–1270 28402133 10.2214/AJR.16.17584

[3] Karliner LS, Patricia Kaplan C, Juarbe T, Pasick R, Pérez-Stable EJ (2005) Poor patient comprehension of abnormal mammography results. J Gen Intern Med 20:432–437 15963167 10.1111/j.1525-1497.2005.40281.x PMC1490112

[4] Yi PH, Golden SK, Harringa JB, Kliewer MA (2019) Readability of lumbar spine MRI reports: will patients understand? AJR Am J Roentgenol 212:602–606 30620671 10.2214/AJR.18.20197

[5] Martin-Carreras T, Cook TS, Kahn CE Jr (2019) Readability of radiology reports: implications for patient-centered care. Clin Imaging 54:116–120 30639521 10.1016/j.clinimag.2018.12.006

[6] Kelly CJ, Karthikesalingam A, Suleyman M, Corrado G, King D (2019) Key challenges for delivering clinical impact with artificial intelligence. BMC Med 17:195 31665002 10.1186/s12916-019-1426-2 PMC6821018

[7] Jeblick K, Schachtner B, Dexl J et al (2024) ChatGPT makes medicine easy to swallow: an exploratory case study on simplified radiology reports. Eur Radiol 34:2817–2825 37794249 10.1007/s00330-023-10213-1 PMC11126432

[8] Schmidt S, Zimmerer A, Cucos T, Feucht M, Navas L (2024) Simplifying radiologic reports with natural language processing: a novel approach using ChatGPT in enhancing patient understanding of MRI results. Arch Orthop Trauma Surg 144:611–618 37950763 10.1007/s00402-023-05113-4

[9] Lyu Q, Tan J, Zapadka ME et al (2023) Translating radiology reports into plain language using ChatGPT and GPT-4 with prompt learning: results, limitations, and potential. Vis Comput Ind Biomed Art 6:9 37198498 10.1186/s42492-023-00136-5 PMC10192466

[10] Salam B, Kravchenko D, Nowak S et al (2024) Generative Pre-trained Transformer 4 makes cardiovascular magnetic resonance reports easy to understand. J Cardiovasc Magn Reson 26:101035 38460841 10.1016/j.jocmr.2024.101035 PMC10981113

[11] Hasani AM, Singh S, Zahergivar A et al (2024) Evaluating the performance of Generative Pre-trained Transformer-4 (GPT-4) in standardizing radiology reports. Eur Radiol 34:3566–3574 37938381 10.1007/s00330-023-10384-x

[12] Salam B, Stüwe C, Nowak S et al (2025) Large language models for error detection in radiology reports: a comparative analysis between closed-source and privacy-compliant open-source models. Eur Radiol. 10.1007/s00330-025-11438-y 10.1007/s00330-025-11438-y PMC12226608 39979623

[13] Bhayana R (2024) Chatbots and large language models in radiology: a practical primer for clinical and research applications. Radiology 310:e232756 38226883 10.1148/radiol.232756

[14] Kim SH, Schramm S, Adams LC et al (2025) Benchmarking the diagnostic performance of open source LLMs in 1933 Eurorad case reports. NPJ Digit Med 8:97 39934372 10.1038/s41746-025-01488-3 PMC11814077

[15] Doshi R, Amin KS, Khosla P, Bajaj SS, Chheang S, Forman HP (2024) Quantitative evaluation of large language models to streamline radiology report impressions: a multimodal retrospective analysis. Radiology 310:e231593 38530171 10.1148/radiol.231593

[16] Nowak S, Biesner D, Layer YC et al (2023) Transformer-based structuring of free-text radiology report databases. Eur Radiol 33:4228–4236 36905469 10.1007/s00330-023-09526-y PMC10181962

[17] Itri JN (2015) Patient-centered radiology. Radiographics 35:1835–1846 26466190 10.1148/rg.2015150110

[18] Park J, Oh K, Han K, Lee YH (2024) Patient-centered radiology reports with generative artificial intelligence: adding value to radiology reporting. Sci Rep 14:13218 38851825 10.1038/s41598-024-63824-z PMC11162416

[19] Ahmed H, Saddouh EA, Abugrin ME et al (2021) Association between patients’ knowledge and adherence to anticoagulants, and its effect on coagulation control. Pharmacology 106:265–274 33202413 10.1159/000511754

[20] Awwad O, Akour A, Al-Muhaissen S, Morisky D (2015) The influence of patients’ knowledge on adherence to their chronic medications: a cross-sectional study in Jordan. Int J Clin Pharm 37:504–510 25708124 10.1007/s11096-015-0086-3

[21] Kianian R, Sun D, Crowell EL, Tsui E (2024) The use of large language models to generate education materials about uveitis. Ophthalmol Retin 8:195–201 10.1016/j.oret.2023.09.008 37716431

[22] Eleyan D, Othman A, Eleyan A (2020) Enhancing software comments readability using Flesch Reading Ease Score. Information 11:430

[23] Nowak S, Wulff B, Layer YC et al (2025) Privacy-ensuring open-weights large language models are competitive with closed-weights GPT-4o in extracting chest radiography findings from free-text reports. Radiology 314:e240895 39807977 10.1148/radiol.240895

[24] Li D, Gupta K, Bhaduri M, Sathiadoss P, Bhatnagar S, Chong J (2025) Comparative diagnostic accuracy of GPT-4o and LLaMA 3-70b: proprietary vs. open-source large language models in radiology. Clin Imaging 118:110382 39740646 10.1016/j.clinimag.2024.110382

[25] Alassan MSY, Espejel JL, Bouhandi M, Dahhane W, Ettifouri EIH (2024) Comparison of open-source and proprietary LLMs for machine reading comprehension: a practical analysis for industrial applications. Preprint at 10.48550/arXiv.2406.13713

[26] Jindal P, MacDermid JC (2017) Assessing reading levels of health information: uses and limitations of Flesch formula. Educ Health (Abingdon) 30:84–88 28707643 10.4103/1357-6283.210517

[27] Kauchak D, Leroy G (2016) Moving beyond readability metrics for health-related text simplification. IT Prof 18:45–51 27698611 10.1109/MITP.2016.50 PMC5044755

[28] van Driel MHE, Blok N, van den Brand JAJG et al (2025) Leveraging GPT-4 enables patient comprehension of radiology reports. Eur J Radiol 187:112111 40294519 10.1016/j.ejrad.2025.112111

[29] Gupta A, Singh S, Malhotra H et al (2025) Provision of radiology reports simplified with large language models to patients with cancer: impact on patient satisfaction. JCO Clin Cancer Inform 9:e2400166 39879570 10.1200/CCI-24-00166

[30] Zhang X, Chowdhury RR, Gupta RK, Shang J (2024) Large language models for time series: a survey. Preprint at 10.48550/arXiv.2402.01801

[31] Akinci D’Antonoli T, Stanzione A, Bluethgen C et al (2024) Large language models in radiology: fundamentals, applications, ethical considerations, risks, and future directions. Diagn Interv Radiol 30:80–90 37789676 10.4274/dir.2023.232417 PMC10916534

[32] Jonnagaddala J, Wong ZS-Y (2025) Privacy preserving strategies for electronic health records in the era of large language models. NPJ Digit Med 8:34 39820020 10.1038/s41746-025-01429-0 PMC11739470

[33] Williamson SM, Prybutok V (2024) Balancing privacy and progress: a review of privacy challenges, systemic oversight, and patient perceptions in AI-driven healthcare. Appl Sci 14:675

[34] Jung K-H (2025) Large language models in medicine: clinical applications, technical challenges, and ethical considerations. Health Inform Res 31:114–124 10.4258/hir.2025.31.2.114 PMC12086438 40384063

[35] Gorenshtein A, Omar M, Glicksberg BS, Nadkarni GN, Klang E (2025) AI agents in clinical medicine: a systematic review. Preprint at 10.1101/2025.08.22.25334232

[36] Farquhar S, Kossen J, Kuhn L, Gal Y (2024) Detecting hallucinations in large language models using semantic entropy. Nature 630:625–630 38898292 10.1038/s41586-024-07421-0 PMC11186750

[37] Li Y, Fu X, Verma G, Buitelaar P, Liu M (2025) Mitigating hallucination in large language models (LLMs): an application-oriented survey on RAG, reasoning, and Agentic Systems. Preprint at 10.48550/arXiv.2510.24476

[38] Bakken S (2023) AI in health: keeping the human in the loop. J Am Med Inform Assoc 30:1225–1226 37337923 10.1093/jamia/ocad091 PMC10280340

[39] Bendi-Ouis Y, Dutartre D, Hinaut X (2024) Deploying open-source large language models: a performance analysis. Preprint at 10.48550/arXiv.2409.14887

[40] Rawte V, Priya P, Tonmoy SMTI, Zaman SMM, Sheth A, Das A (2023) Exploring the relationship between LLM hallucinations and prompt linguistic nuances: readability, formality, and concreteness. Preprint at 10.48550/arXiv.2309.11064

[41] Asgari E, Montaña-Brown N, Dubois M et al (2025) A framework to assess clinical safety and hallucination rates of LLMs for medical text summarisation. NPJ Digit Med 8:274 40360677 10.1038/s41746-025-01670-7 PMC12075489

[42] OpenAI, Achiam J, Adler S et al (2023) GPT-4 technical report. Preprint at 10.48550/arXiv.2303.08774

[43] Ali M, Fromm M, Thellmann K et al (2024) Tokenizer choice for LLM training: negligible or crucial? In: Findings of the Association for Computational Linguistics: NAACL 2024. Association for Computational Linguistics, Mexico City, Mexico, pp 3907–3924

