# Supplementary material for: Simplifying radiology reports with large language models: privacy-compliant open- versus closed-weight models
Source: Eur Radiol. 2026 Feb 12;36(7):6097–106. doi: 10.1007/s00330-026-12329-6 (PMC13282196; doi:10.1007/s00330-026-12329-6)
Supplement: Supplementary file 1 — Supplementary information [file 330_2026_12329_MOESM1_ESM.pdf]

Simplifying Radiology Reports With Large Language Models: Privacy-Compliant Open Versus Closed-weight Models

ELECTRONIC SUPPLEMENTARY MATERIAL

Supplement S1 Text shows the full prompt to simplify radiology reports. The English renderings were produced by a human translator (native-speaking author).

You are a radiology specialist with over 20 years of professional experience and you have just written the following findings for an imaging procedure. Now create a radiological report based on these findings that is understandable for the patient, who is a medical layperson. Explain all medical terms and abbreviations in such a way that a layperson understands them. Explain them the first time they are mentioned and also explain already known diseases or findings. This report should have the following 5 sections: Examination, Why the examination was done, Findings, Simple explanation, and Impression. No greeting or farewell is desired. Use a professional, polite, and understandable communication style. Address the patient in the second-person pronoun “you” form. Refer to yourself in the first-person plural form. The language should be adapted to a reading level suitable for someone with an eighth-grade education.

Supplement S2 Exemplary CT radiology report A original report, B Mixtral-8x22B generated report, C GPT-4o generated report, D Llama-3-70b generated report. The English renderings were produced by a human translator (native-speaking author).

(A)

Examination: Non-contrast CT of the head and midface

Indication: Fall on face. Evaluate for post-traumatic sequelae and possible left orbital fracture.

Impression: Dislocated fracture of the left orbital roof extending into the left frontal sinus. No ICB.

(B)

Examination: You have had a computerised tomography (CT) scan of the skull and midface. This is a special type of X-ray examination that produces detailed images of your head.

Why the scan was done: The scan was done because you had a fall on your face. We wanted to check if there were any injuries or damage to your face and head as a result of the fall. In particular, we wanted to find out if there was a fracture (broken bone) in the left eye socket (orbit).

Findings: The examination revealed that you have a dislocated (displaced) fracture of the left orbital roof. This means that part of the bone that forms the upper half of your left eye socket is broken and displaced. In addition, this fractured bone has penetrated into the left frontal sinus. However, no intracranial haemorrhage (ICB), i.e. bleeding in the brain, was detected.

Simple explanation: In simple language, this means that you have a broken bone in your left eye socket caused by the fall. The broken bone is slightly displaced and has moved into the left frontal sinus. Fortunately, there is no hemorrhage in your brain.

Impression: Based on this examination, we recommend that you see a specialist to plan further treatment for your fracture. It is important that you follow your doctor's instructions to ensure a full recovery.

(C)

Examination:

We have carried out a computerised tomography (CT) scan of your head and midface. A CT scan uses X-rays to take detailed pictures of the inside of your body.

Why the scan was done:

You fell on your face. As we wanted to check whether the fall had caused any injuries, in particular whether you had a fracture in the area of your eye socket (orbital fracture) on the left side, we carried out this examination.

Findings:

We found that there is a displaced fracture in the area of the roof of your left eye socket. This fracture also extends into the left frontal sinus. However, there are no signs of bleeding in the brain (intracranial haemorrhage, ICB for short).

Simple explanation:

During your fall, a bone in the upper part of the left eye socket fractured out of place and penetrated into the left frontal sinus. However, there is no haemorrhage in the brain, which is good news.

Impression:

The fracture in the area of the left eye socket must be treated. Surgery may be required to reset the bones correctly. It is important that you take it easy over the next few days and weeks and follow all your doctor's instructions to ensure the best possible healing. Fortunately, no other serious injuries, such as bleeding in the brain, have occurred.

(D)

Examination:

We have performed a computerised tomography (CT) scan of the skull and midface without using contrast media. This examination is also referred to as "native".

Why the examination was carried out:

You came to our clinic after a fall on your face and we wanted to determine whether there were any injuries to the face and skull. In particular, we wanted to clarify whether there had been a fracture (broken bone) in the area of the left orbital rim.

Findings:

We found a dislocated fracture (a bone fracture in which the bone fragments are no longer in their normal position) of the left orbital roof. This orbital roof is a part of the skull that surrounds the eyeball. The fracture has led to a collapse of the left frontal sinus. The frontal sinus is an air pocket in the skull located above the eyes. We could not detect any intracranial haemorrhage (ICB), i.e. there is no bleeding in the brain.

Simple explanation:

You have a bone fracture in the area of the left orbital rim, which has led to a herniation into the left frontal sinus. Fortunately, there is no haemorrhage in the brain.

Impression:

We recommend further treatment by a specialist in oral and maxillofacial surgery to treat the fracture and minimise any possible consequences. We will provide you with further information about the next steps and treatment options.

Supplement S3 Exemplary MRI radiology report A original report, B Mixtral-8x22B generated report, C GPT-4o generated report, D Llama-3-70b generated report.

(A)

Examination: MRI heart with CM

Indication: Cardio-MRI in case of a post-COVID infection 4 weeks ago, since then global cardiac decompensation with new-onset left heart failure (EF 47%). Presence of Covid-induced myocarditis.

Impression: No evidence of (post-) inflammatory myocardial changes. Biventricular global hypokinesia with mild to moderately impaired left ventricular pump function (LVEF 46%) with normal ventricular dimensions (LVEDV/BSA 83 ml/m²). Mild to moderate aortic valve insufficiency, mild tricuspid valve insufficiency.

(B)

Examination:

You have had an MRI examination of the heart with contrast medium (CM). An MRI scan is an imaging procedure that makes it possible to obtain detailed images of the heart and its functions.

Indication:

The examination was performed because you contracted a Covid infection 4 weeks ago and have since suffered from global cardiac decompensation with newly developed left heart failure (EF 47%). Global cardiac decompensation means that the heart is no longer able to pump enough blood through the body. Left heart failure refers to a weakness of the left heart to pump blood around the body.

The EF (ejection fraction) is a measure of the heart's pumping function. An EF of 47% means that the heart is only actually pumping about half the normal volume of blood it should be pumping.

It was suspected that the Covid infection triggered an inflammation of the heart muscle (myocarditis).

Impression:

We found no signs of a (post-)inflammatory change in the heart muscle. Global hypokinesia of both ventricles (biventricular) means that the ventricles can only contract and pump to a limited extent. The left ventricular pumping function (LVEF) is slightly to moderately restricted (LVEF 46%), which means that the heart is only pumping around half of the normal blood volume. However, the ventricular dimension (LVEDV/BSA 83 ml/m²) is normal.

Low to moderate aortic valve insufficiency was found, which means that the heart valve between the aorta and the left ventricle does not close properly and blood may flow backwards. Similarly, mild tricuspid regurgitation was found, which means that the valve between the right ventricle and the right atrial chamber does not close properly and blood may flow backwards.

Simple explanation:

We have found no evidence that the Covid infection has caused inflammation of the heart muscle. However, your heart is only pumping about half the normal blood volume, which leads to left heart failure. However, the ventricular dimension is normal. There is also mild to moderate aortic valve insufficiency and mild tricuspid valve insufficiency.

Impression:

Overall, the results of the examination are reassuring, as no signs of Covid-induced myocarditis were found. However, it is important to monitor left heart failure closely in the future and treat it if necessary.

(C)

Examination:

We have carried out a magnetic resonance imaging (MRI) scan of your heart using a contrast medium (CM).

Why the examination was carried out:

The examination was carried out because you were diagnosed with Covid-19 four weeks ago. Since then, you have noticed a general deterioration in your heart function (global cardiac decompensation) and a new weakness in the left side of your heart (left heart failure). Your heart is pumping less effectively than normal (ejection fraction, EF for short, is 47%). We suspected inflammation of the heart muscle (myocarditis) as a result of the Covid infection.

Findings:

No signs of a (post-)inflammatory change in the heart muscle were detected. Your heart shows restricted movement of both ventricles (biventricular global hypokinesia) and the pumping function of the left ventricle is slightly to moderately restricted (left ventricular ejection fraction, LVEF, is 46%). However, the size of the ventricles is normal. There is also a slight to moderate leak in the aortic valve (aortic valve insufficiency) and a slight leak in the tricuspid valve.

Simple explanation:

Your heart has no inflammation due to your Covid infection. Both ventricles are moving less than they should and the pumping capacity of the left ventricle is slightly to moderately reduced. The size of your ventricles is normal. There is mild to moderate leakage in one heart valve (aortic valve) and mild leakage in another heart valve (tricuspid valve).

Impression:

To summarise, your heart shows no inflammation, but reduced movement and pumping capacity. The size of your heart is normal. The detected leaks in the heart valves are mild to moderate and should be monitored. We recommend regular monitoring and, if necessary, adjusting your treatment to support heart function and alleviate symptoms. Please discuss the next steps with your doctor.

(D)

Examination:

We carried out a magnetic resonance imaging (MRI) procedure on the heart using a contrast medium (CM). This procedure allows us to create detailed images of the heart.

Why the examination was carried out:

The scan was carried out because you had a Covid-19 infection four weeks ago and have had problems with your heart since then. Your heart is struggling to pump enough blood around the body, leading to global cardiac decompensation. In particular, you have new-onset left heart failure, which means that the left ventricle can no longer pump blood as effectively. We wanted to investigate whether the Covid-19 infection has caused inflammation in the heart tissue.

Findings:

Our examination has shown that there are no signs of inflammation in the heart tissue. However, we found that both ventricles (left and right) are not pumping as effectively as they should. The left ventricle is only pumping about 46% of the blood it would normally pump. However, the size of the ventricles is normal. We also found that the aortic valve and the tricuspid valve, the two main valves of the heart, have mild to moderate problems letting blood through.

Simple explanation:

The good news is that we found no signs of inflammation in the heart tissue. However, we did find that your heart is not pumping as effectively as it should. This can lead to problems if your heart can't pump enough blood around the body. We also found that the valves of the heart have slight problems letting blood through.

Impression:

We will discuss these results with your treating doctor to decide what next steps are needed to improve your heart health. We will also carry out further investigations to monitor and treat the degree of heart failure.

Supplement S4 Exemplary X-ray radiology report, A original report, B Mixtral-8x22B generated report, C GPT-4o generated report, D Llama-3-70b generated report.

(A)

Examination: X-ray cervical spine in two planes, X-ray lumbar spine in two planes, X-ray thoracic spine in two planes

Indication: After an unobserved fall, spinal knocking pain. Fracture?

Impression: Limited assessability in osteoporosis. Constant visualisation of the previously known sintering of BWK 9 and LWK 1-3 without interim post-sintering. As far as can be assessed, no fresh fractures.

(B)

Examination:

You have had an X-ray examination of the cervical spine (cervical spine), the lumbar spine (lumbar spine) and the thoracic spine (thoracic spine) in two planes. The cervical spine is the upper part of the spine that supports your head. The thoracic spine is the middle part that supports your ribs. The lumbar spine is the lower part of the spine that carries most of the weight of your body.

Indication:

The examination was carried out because you complained of spinal tapping pain after an unobserved fall. Spinal knocking pain is pain in the back that can be triggered by knocking on the bones of the spine. The question was whether you may have sustained a fracture.

Findings:

The spine can only be assessed to a limited extent due to osteoporosis. Osteoporosis is a disease in which the bones become brittle and can break more easily. A constant visualisation of the previously known sintering of BWK 9 and LWK 1-3 without interim post-sintering was determined.

Sintering is a bone fracture that has already healed. BWK 9 and LWK 1-3 refer to the thoracic spine and the lumbar spine. As far as could be assessed, no fresh fractures (breaks) were found.

Explanation:

There is osteoporosis, which limits the ability to assess the spine. No new fractures were found, but the already known fractures of the thoracic spine and lumbar spine remained stable and did not worsen.

Impression:

No new fractures were found. The already known sintering has remained stable. We recommend that you talk to your doctor about the options for treating osteoporosis in order to reduce the risk of further fractures.

(C)

Examination:

We have carried out an X-ray examination of your cervical spine (cervical spine), thoracic spine (thoracic spine) and lumbar spine (lumbar spine) from two different angles. This means that we took pictures of your spine from different perspectives.

Why the examination was carried out:

The examination was done because you had an unobserved fall and felt pain in your spine when it was tapped. We wanted to check whether there were any fractures in your spine.

Findings:

Our examination shows that it is more difficult to make precise statements due to existing osteoporosis (bone atrophy in which the bones become thinner and weaker).

However, we can see that the known sintering (indentations of the vertebral bodies caused by previous compression) on the vertebrae of the thoracic spine (BWK 9) and the lumbar spine (LWK 1-3) are unchanged. This means that these old injuries have not worsened. We could not recognise any new fractures.

Simple explanation:
During the X-ray examination of your spine, we saw that your old bone injuries have not changed and there are no new fractures. Your bones are weakened by osteoporosis, which made the examination somewhat more difficult.

Impression:
Overall, this is good news: your spine has no new fractures and your old injuries have remained stable. However, it would be important to continue to treat the osteoporosis and take measures to prevent further fractures.

(D)

Examination:
We have performed an X-ray procedure to examine your spine. We took X-ray images in two planes of your cervical spine (cervical spine), your lumbar spine (lumbar spine) and your thoracic spine (thoracic spine).

Why the examination was carried out:
The examination was carried out because you had an unexpected fall and since then have had pain in your back that feels like a knocking. We wanted to find out if you might have a fracture (broken bone).

Findings:

When we analysed the x-rays, we found that the Impression was difficult because you have osteoporosis. Osteoporosis is a disease in which the bones become weak and porous. We also saw that the previously known sintering (small bone fractures) on vertebral bodies 9 of the thoracic spine (BWK 9) and vertebral bodies 1-3 of the lumbar spine (LWK 1-3) are still present. Fortunately, we were unable to detect any new, fresh fractures.

Simple explanation:

This means that we have not found any new fractures due to the fall. The pain you are experiencing could be caused by the previous fractures or osteoporosis.

Impression:

We recommend that you see a doctor for further treatment of the pain and osteoporosis. It is important that you continue your osteoporosis treatment to prevent further fractures. We are here for you if you have any further questions or require further investigations.

Supplement S5 Exemplary ultrasound radiology report, A original report, B Mixtral-8x22B generated report, C GPT-4o generated report, D Llama-3-70b generated report.

(A)

Examination: Ultrasound of right sided cervical lymph nodes.

Indication: Swelling in the nuchal area on the right that has been present for 3 weeks. Entity of the swelling? Lymph node? Malignancy suspected? Abnormalities?

Impression: Small subcutaneous lymph node without imaging features suggestive of malignancy.

(B)

Examination:

You have undergone an ultrasound examination of the neck and the tissue to the right under the skin (subcutis). An ultrasound is a non-invasive, painless examination in which we use sound waves to produce images of internal organs or tissues.

Why the examination was performed:

The examination was performed because you have had swelling in the right side of your neck for three weeks. The term "nuchal" refers to the neck area. We carried out the examination to find out what type of swelling it was. There was a suspicion that it could be an enlarged lymph node or even a malignant growth (malignoma).

Findings:

We found a small lymph node under the skin (subcutaneous) on the right side. This lymph node shows no signs of being malignant (malignancy criteria).

Simple explanation:

A lymph node is a small organ that is part of the immune system. It helps fight infection and disease. When you are ill or have an infection, the lymph nodes can become enlarged.

However, in your case, the lymph node is not so large that we should be concerned. Furthermore, there are no signs that it is something serious like cancer.

Impression:

In our Impression, the swelling you have in the right side of your neck is nothing to worry about. However, we recommend that you continue to be monitored by us to ensure that the swelling does not increase further or show signs of anything serious.

(C)

Examination:

We carried out an ultrasound examination of the neck on the right side. An ultrasound is an imaging procedure that uses sound waves to produce images of the internal structures of the body.

Why the examination was carried out:

You have had swelling in your neck on the right side for three weeks. We wanted to find out what was causing this swelling. In particular, we wanted to know whether it was an enlarged lymph node, whether there was any suspicion of cancer (suspected malignancy) or whether there were any other abnormalities.

Findings:

We found a small lymph node under the skin. This lymph node shows no signs that would indicate cancer.

Simple explanation:

A lymph node is a small, bean-shaped lump that is part of your immune system and helps fight infection. Sometimes lymph nodes can swell when you have an infection or inflammation.

In your case, the lymph node is small and shows no features that would indicate cancer. There are no other abnormal findings.

Impression:

The swelling in your neck is most likely caused by an enlarged lymph node that shows no signs of malignancy. This is a good sign. We recommend that you continue to monitor the lump. If the swelling does not go down or if new symptoms appear, you should seek medical advice again.

(D)

Examination:

We performed an ultrasound of the neck and subcutaneous tissue on the right side. An ultrasound is an imaging procedure that helps us to examine internal structures of the body without X-rays or other harmful substances.

Why the examination was carried out:

You have noticed swelling in the area of the neck (nuchal) on the right side over the last three weeks. We wanted to find out what was causing this swelling and whether it was possibly a lymph node or a malignant tumour (malignoma).

Findings:

We found a small lymph node located in the subcutaneous tissue of the neck on the right side. A lymph node is a part of the immune system that helps protect the body from infection. In this case, we see no signs that the lymph node is malignant.

Simple explanation:

The swelling you have noticed is caused by a small lymph node. This is a normal part of the immune system and not a cause for concern. We could find no evidence that the lymph node is malignant.

Impression:

We are relieved that we found no evidence of a malignant tumour. The lymph node itself is very small and does not appear aggressive. We do not recommend any further investigations or treatment in this case. However, if you have any further questions or concerns, you should contact your doctor.
